# SRC-3 deficiency prevents atherosclerosis development by decreasing endothelial ICAM-1 expression to attenuate macrophage recruitment

**DOI:** 10.7150/ijbs.74864

**Published:** 2022-10-03

**Authors:** Wenbo Chen, Wuyang Zheng, Shixiao Liu, Qiang Su, Kangxi Ding, Ziguan Zhang, Ping Luo, Yong Zhang, Jianming Xu, Chundong Yu, Weihua Li, Zhengrong Huang

**Affiliations:** 1 Department of Cardiology, Xiamen Key Laboratory of Cardiac Electrophysiology, Xiamen Institute of Cardiovascular Diseases, The First Affiliated Hospital of Xiamen University, School of Medicine, Xiamen University, Xiamen, China.; 2 Key Laboratory of Prevention and treatment of cardiovascular and cerebrovascular diseases of Ministry of Education, Jiangxi Provincial Clinical Research Center for Vascular Anomalies, Gannan Medical University, Ganzhou, Jiangxi, China.; 3 State Key Laboratory of Cellular Stress Biology, Innovation Center for Cell Biology, School of Life Sciences, Faculty of Medicine and Life Sciences, Xiamen University, Xiamen, China.; 4 Department of Molecular and Cellular Biology, Baylor College of Medicine, Houston, TX, USA.

**Keywords:** SRC-3, endothelial cell, ICAM-1, bufalin, NF-κB signaling

## Abstract

Steroid receptor coactivator 3 (SRC-3) is a member of the p160 SRC family. This factor can interact with multiple nuclear hormone receptors and transcription factors to regulate the expression of their target genes. Although many physiological roles of SRC-3 have been revealed, its role in atherosclerosis is not clear. In this study, we found that SRC-3^-/-^ApoE^-/-^ mice have reduced atherosclerotic lesions and necrotic areas in their aortas and aortic roots compared with SRC-3^+/+^ApoE^-/-^ mice after Western diet (WD) feeding for 12 weeks. RNA-Seq and Western blot analyses of the aorta revealed that SRC-3 was required for maintaining the expression of ICAM-1, which was required for macrophage recruitment and atherosclerosis development. siRNA-mediated knockdown of SRC-3 in endothelial cells significantly reduced WD-induced atherosclerotic plaque formation. Additionally, treatment of ApoE^-/-^ mice with SRC-3 inhibitor bufalin prevented atherosclerotic plaque development. SRC-3 deficiency reduced aortic macrophage recruitment. Accordingly, ICAM-1 expression was markedly decreased in the aortas of SRC-3^-/-^ApoE^-/-^ mice and ApoE^-/-^ mice with endothelial SRC-3 knockdown mediated by AAV9-shSRC-3 virus. Mechanistically, SRC-3 coactivated NF-κB p65 to increase *ICAM-1* transcription in endothelial cells. Collectively, these findings demonstrate that inhibiting SRC-3 ameliorates atherosclerosis development, at least in part through suppressing endothelial activation by decreasing endothelial ICAM-1 expression via reducing NF-κB signaling.

## Introduction

Atherosclerosis is a chronic inflammatory disease of the large arterial walls and is one of the main causes of death worldwide 1. The pathology of atherosclerosis is characterized by the subendothelial accumulation of inflammation, vascular wall cells, extracellular matrix, and apolipoprotein B-containing lipoproteins 2. The entry and retention of apolipoprotein B containing lipoproteins into the intima is a key initiating event in atherosclerosis 3. The activation of endothelial cells can increase the expression of leukocyte adhesion molecules (VCAM-1 and ICAM-1) and chemoattractants (MCP-1), promoting the entry of bone marrow-derived monocytes into the intima, where these cells differentiate into macrophages 4, 5. Macrophage internalizes oxLDL through CD36 and SR-1 to form foam cell, which leads the formation of atheromas and plays a critical role in plaque initiation and development 6, 7. The typical pathology of vulnerable plaques includes large areas of necrotic cores and thinning of the fibrous cap. Plaque rupture of the fibrous cap leads to thrombus formation, which is the primary contributor to cardiovascular events, such as stroke, myocardial infarction, and sudden cardiac death 8. Therefore, understanding the underlying molecular mechanisms can provide information for the development of effective preventive and therapeutic strategies.

Steroid receptor coactivator 3 (SRC-3) belongs to the p160 coactivator family, which interacts with multiple nuclear receptors and other transcription factors to enhance their effects on target gene transcription 9. Our previous studies showed that SRC-3 expression in macrophages could protect against LPS-induced endotoxic shock by regulating the proinflammatory cytokine balance 10 and protect against *Escherichia coli*-induced septic peritonitis by enhancing the phagocytosis of bacteria and reducing macrophage apoptosis 11. Our recent study demonstrated that SRC-3^-/-^ mice displayed delayed clearance of *Citobacter rodentium* and more severe tissue pathology after oral infection with *C. rodentium* due to decreased production of the chemokines CXCL2 and CXCL5 and decreased recruitment of neutrophils, indicating that SRC-3 plays a critical protective role in bacteria-induced colitis 12. It has been reported that SRC-3 has an ER-dependent vasoprotective role in vascular trauma by suppressing vascular cell proliferation 13, and deletion of SRC-1/3 causes noncompaction cardiomyopathy in the hearts of newborn and adult mice by inhibiting cardiomyocyte proliferation and differentiation 14, suggesting that SRC-3 has a protective role in cardiovascular; however, the biological role of SRC-3 in atherosclerosis remains poorly defined.

In this study, we used SRC-3^-/-^ApoE^-/-^ mice to assess the role of SRC-3 in atherosclerosis. We found that SRC-3^-/-^ApoE^-/-^ mice exhibited fewer atherosclerotic lesions and necrotic areas in the aortas and aortic roots than SRC-3^+/+^ApoE^-/-^ mice after being fed a high-fat diet for 12 weeks. Furthermore, knockdown of SRC-3 in endothelial cells ameliorated atherosclerosis development. SRC-3 accelerated atherosclerosis development, at least in part through increasing endothelial adhesion and the transmigration of leukocytes by promoting ICAM-1 expression in endothelial cells via enhancing NF-κB function.

## Materials and methods

### Mice and Diets

SRC-3^-/-^ mice on a C57BL/6×129Sv background 15 were bred with the ApoE^-/-^ mice on a C57BL/6J background (a gift from Jiahuai Han, Xiamen University) to generate SRC-3^+/-^ApoE^-/-^ mice. Male SRC-3^+/+^ApoE^-/-^ and SRC-3^-/-^ApoE^-/-^ mice (8 weeks old) were fed a WD for 12 weeks. The WD was produced by Vital River Laboratories (Beijing, China; fed to SRC-3^-/-^ApoE^-/-^ mice and mice that were injected with AAV-shSRC-3 or AAV-hICAM-1) or Ready Biotechnolgy (Shenzhen, China; fed to mice that were injected with AAV-shICAM-1) according to the protocol from Harlan (TD.88137). Animal experiments were approved by the Laboratory Animal Center of Xiamen University, Xiamen, Fujian, China.

### Human samples

Human lower limb aortic tissue specimens were obtained from 16 patients with arteriosclerosis obliterans who had undergone surgery at Gannan Medical University. Written informed consent was obtained from all individuals, and the study protocol was approved by the Institute Research Ethics Committee at the First Hospital of Xiamen University and Gannan Medical University.

### Cell culture and transient transfection

HUVECs (purchased from National Stem Cell Translational Resource Center, mycoplasma negative, DFSC-EC-01) were maintained in EGM2 media (Lonza) supplemented with 10% fetal bovine serum (Gibco) and the EGM-2 bullet kit at 37 °C and 5% CO_2_. After reaching 60% confluence, the HUVECs were transfected with SRC-3 siRNA (Genepharma, Shanghai) or scrambled siRNA by using Lipofectamine 2000 (Invitrogen) according to the manufacturer's instructions. The cells were cultured for 48 h or 72 h before further experiments.

### *En face* analysis of atherosclerosis and plaque histology

For *en face* analysis of atherosclerotic lesions, the whole aorta was removed and stained with Oil Red O, and the lesion areas were quantified with Image-Pro Plus 6.0 (Image Metrology, Copenhagen, Denmark). Hearts were fixed in 4% paraformaldehyde and embedded in paraffin or O.C.T. for histological analysis. Five-micrometer sections of the atrioventricular valve region of the heart were cut and stained with hematoxylin and eosin (H&E) to analyze atherosclerosis lesions or Masson reagents to analyze collagen contents. Atherosclerotic lesions were analyzed in six consecutive sections from 4-6 different littermates in each group using Image-Pro Plus 6.0. The plaque stability score = (α-SMC area + collagen area) / (macrophage area + lipid area) 16.

### Immunohistochemistry

Five-micrometer consecutive sections of aortic roots were cut, deparaffinized and rehydrated. Antigens were retrieved by soaking in citrate buffer (pH 6.0) with microwave heating for 20 min. The sections were blocked in 10% goat serum for 30 min and incubated with the following primary antibodies overnight: anti-F4/80 (Cell Signaling Technology, D4C8V, #30325) and anti-α-smooth muscle actin (Abcam, E184, ab32575). The sections were incubated with 3% H_2_O_2_ for 10 min at the room temperature to eliminate endogenous peroxidase activity and then incubated with Elivision Plus kits (Maixin) at room temperature. DAB reagent was used to visualize the stained proteins. The positive areas were analyzed with Image Pro-Plus 6.0.

### RNA-sequencing analysis

Total RNA samples were isolated from the aortas of SRC-3^-/-^ApoE^-/-^ and SRC-3^+/+^ApoE^-/-^ mice using TRIZOL reagent (Invitrogen) and treated with RNase-free DNase I. The extracted RNA samples were sent to GENEWIZ for RNA-sequencing analysis. Each sample represented a pooled sample of three representative mice. *P*<0.05 and fold change>1.5 were defined as the thresholds for significantly differential gene expression. Pathway enrichment analysis and GO analysis were based on the KEGG pathway database and Gene Ontology Database, respectively.

### Endothelial cell-enhanced AAV packaging

The shuttle plasmids were cotransfected into HEK-293T cells with endothelial cell-enhanced RGDLRVS-AAV9-cap plasmids (a gift from O. J. Müller, Universitat Heidelberg, Germany) and pHelper 17. The AAV viral particles were isolated and purified according to a previously reported protocol 18. For AAV-mediated ICAM-1 overexpression and SRC-3 and ICAM-1 knockdown, 1 × 10^10^ viral genomes were injected into male 8-week-old ApoE^-/-^ mice via the tail vein before the mice were fed a WD.

### Oral administration of bufalin in ApoE^-/-^ mice

For the preservation model, 8-week-old male ApoE^-/-^ mice were intraperitoneally injected with 1.0 mg/kg bufalin 6 times per week (once daily) 19 for 13 weeks and were fed a WD. For the regression model, 8-week-old male ApoE^-/-^ mice were fed a WD for 10 weeks followed by 1.0 mg/kg bufalin administration 6 times per week (once daily) for another 13 weeks while being fed a WD. DMSO containing 0.9% NaCl was used as a vehicle. The mice were killed and dissected. Oil Red O staining was used to determine atherosclerotic plaque formation.

### Quantitative real-time PCR

Total RNA was extracted from tissues and cells using TRIzol reagent (Invitrogen). RNA integrity was assessed by absorbance spectroscopy, and samples with an OD260/OD280 of approximately 1.9 were used for experiments. One microgram of total RNA was reverse transcribed using a ReverTra Ace Qpcr RT Master Mix kit (TOYOBO). Real-time PCR was performed using FastStart Universal SYBR Green Master Mix (Rox) (Roche). The relative mRNA level was calculated by normalization to GAPDH. The primer sequences are available upon request.

### Measurement of plasma lipids and glucose

Total cholesterol and high-density lipoprotein levels were measured by kits (Nanjing Jiancheng) according to the manufacturer's instructions. Blood glucose levels were measured by a glucometer (Roche).

### Immunoblot analysis

HUVECs and the whole aorta were lysed in RAPA buffer (150 mM NaCl, 50 mM Tris, 0.1% SDS, 1 mM EDTA, 1% Triton X-100, 1 mM PMSF and protease inhibitors). Proteins were quantified with a BCA assay. Equal amounts of proteins were loaded onto 8% sodium dodecyl sulfate-polyacrylamide gel electrophoresis gels and transferred onto polyvinylidene difluoride membranes (Millipore), followed by immunoblotting with anti-SRC-3 (Cell Signaling Technology, 5E11, #2116), anti-human ICAM-1 (Abcam, EPR4776, ab109361), anti-mouse ICAM-1 (Abcam, EPR16608, ab179707), anti-p65 (Cell Signaling Technology, D14E12, #8284), anti-p-p65 (Cell Signaling Technology, Ser536, #3031), anti-GAPDH (Cell Signaling Technology, D16H11, #5174) and anti-β-actin (Sigma, AC-15, #A5441). Western blots were analyzed using a Tonen Image System.

### Construction of plasmids and the luciferase reporter assay

Full-length human ICAM-1 cDNA was amplified by polymerase chain reaction (PCR) from HUVECs with the following primer sets: forward, ICAM-1 5'-CCGGAATTCATGGCTCCCAGCAGCCC-3'; reverse, ICAM-1 5'-TGCTCTAGATCAGGGAGGCGTGGCTTGTG-3' and then inserted into the pCR3.1-HA vector at the *EcoRI* and *XbaI* sites to yield the human ICAM-1 expression plasmid. The human ICAM-1 promoter 1007-bp fragment was amplified by PCR using mouse genomic DNA as a template with the following primer sets: forward, ICAM-1 (-1092) 5'-CGGGGTACCCTTAAGAGTACCCAGCCTCGAC-3'; reverse, ICAM-1 (-88) 5'-CGACGCGTCCCCTCCGGAACAAATGCT-3'. The fragment was ligated into the *KpnI* and *MluI* sites of the luciferase reporter vector PGL3-basic to produce the ICAM-1 promoter reporter plasmid. To generate site mutations in NF-κB, the transcription factor recognition site was abrogated by a one-step site-directed and site-saturation mutagenesis method 20. The primers for site-directed mutation were yielded as follows: mNF-κB, forward, 5'-TaagAATTCCGGAGCTGAAGCGGCCAGCG-3', reverse, 5'-TCAGCTCCGGAATTcttAAGCTAAAGCAAT-3'. Lowercase letters indicate mutated sites. DNA sequencing was used to verify the nucleotide sequences of these constructs. Luciferase activity was examined by a Dual Luciferase Reporter Assay System (Promega, Madison, WI). *Renilla* luciferase activity was used to normalize the transfection efficiency.

### Chromatin immunoprecipitation assay

Wild-type HUVECs or transient SRC-3-knockdown HUVECs were used for chromatin immunoprecipitation (ChIP) assays, which were performed according to the method described by Abcam (Cambrige, MA). The following primers were used: ICAM-1 promoter NF-κB binding site, forward, 5'-GTCATCGCCCTGCCACC-3' and reverse, 5'-ATTTCCGGACTGACAGGGTG-3'. Anti-SRC-3 antibodies (C-20, sc-7216) and anti-p65 (D14E12, #8284) antibodies were purchased from Santa Cruz Biotechnology and Cell Signaling Technology, respectively.

### Monocyte-endothelial adhesion and transendothelial migration assays

For the monocyte-endothelial adhesion assay, confluent HUVECs were transfected with scramble or SRC-3 siRNA for 48 h and then the scramble or SRC-3 siRNA-transfected HUVECs were stimulated with 10 ng/ml TNF-α or 2 ng/ml IL-1β for 3 h or 6 h. After 3 h or 6 h, Calcein (Yeasen)-labeled THP-1 monocytes were cocultured with the stimulated HUVECs for 30 min followed by two rounds of gentle washing to remove nonadherent THP-1 cells, and the cells were fixed and imaged with fluorescence microscopy at 488 nm. At least five random fields were imaged to quantify adherent THP-1 cells per condition.

For the transendothelial migration assay, confluent HUVECs were transfected with scramble or SRC-3 siRNA for 24 h, and then the scramble or SRC-3 siRNA-transfected HUVECs were seeded onto 6.5 mm Transwell filters coated with 0.1% gelatin (Sigma-Aldrich) for another 24 h. The HUVECs were stimulated with 10 ng/ml TNF-α or 2 ng/ml IL-1β for 6 h followed by the addition of calcein-labeled THP-1. After being incubated for 24 h, the transmigrated THP-1 cells were imaged and quantified.

### Statistical analysis

Data were collected from at least two independent experiments. All data are expressed as the mean ± SEM. Statistical significance was determined by unpaired two-tailed Student's t test. *P*<0.05 was considered statistically significant.

## Results

### SRC-3 deficiency prevents the development of atherosclerosis and enhances the stability of atherosclerotic plaques

**Figure 1:**
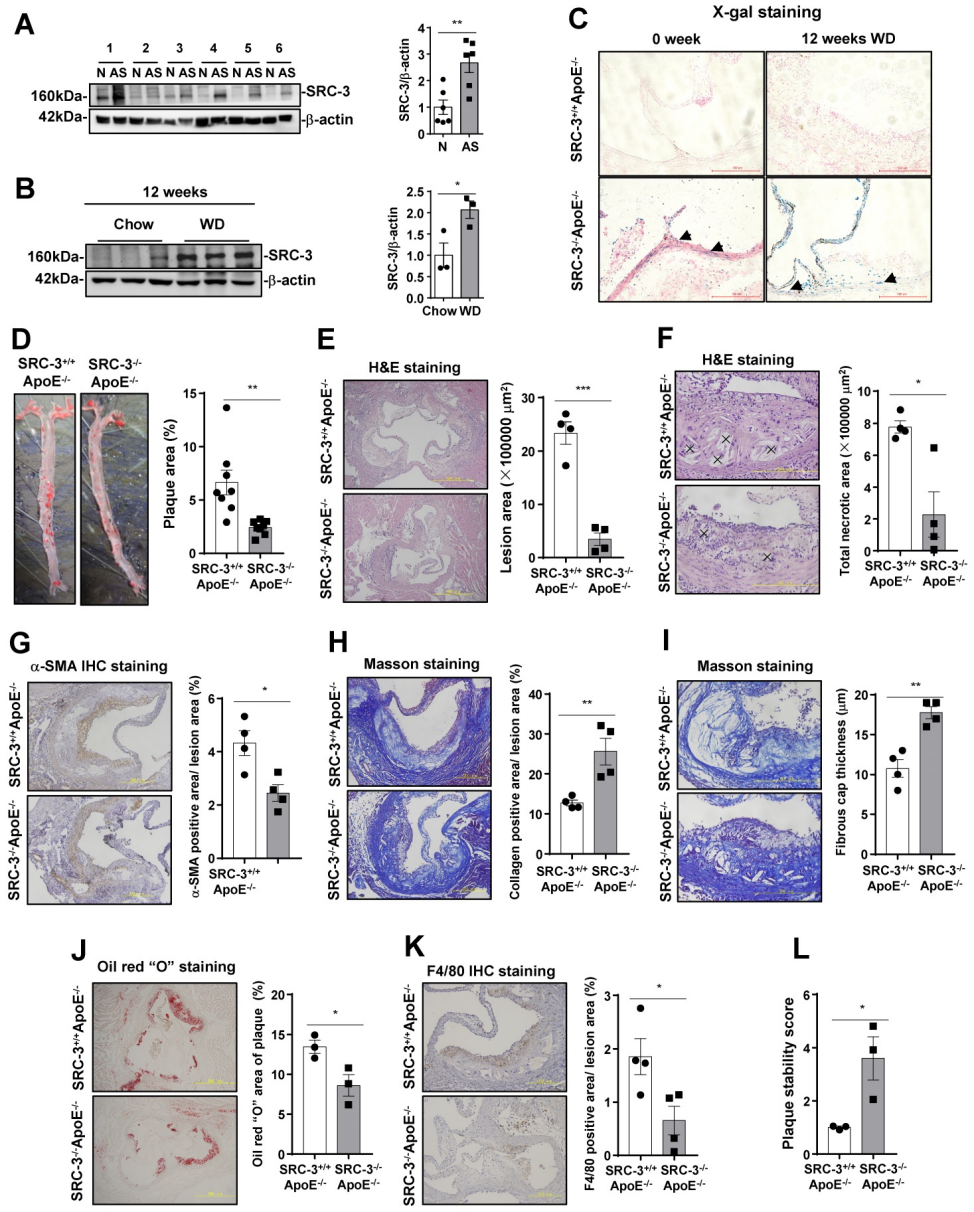
** SRC-3^+/+^ApoE^-/-^ mice exhibit more severe atherosclerosis. (A)** SRC-3 was highly expressed in human atherosclerotic plaques. N represents plaque-adjacent vasculature in the lower limb aorta. AS represents atherosclerotic plaques in the lower limb aorta. **(B)** SRC-3 expression was upregulated in the aortas of ApoE^-/-^ mice after the mice were fed a WD for 12 weeks. **(C)** SRC-3 was expressed in the endothelial cells and vascular smooth muscle cells of chow-fed SRC-3^-/-^ApoE^-/-^ mice and was further increased after the mice were fed a WD for 12 weeks. Sections of frozen aortic roots from SRC-3^+/+^ApoE^-/-^and SRC-3^-/-^ApoE^-/-^ mice were subjected to X-gal staining. Arrows indicate positively stained cells (blue). Scale bar, 100 µm. **(D)** SRC-3 promoted atherosclerotic plaque formation. Representative images of *en face* Oil Red O-stained aortas from SRC-3^+/+^ApoE^-/-^ and SRC-3^-/-^ApoE^-/-^ mice (left panel). Quantification of the plaque areas in aortas (right panel). **(E-K)** Cross-sections of the aortic roots from SRC-3^+/+^ApoE^-/-^ and SRC-3^-/-^ApoE^-/-^ mice were subjected to (E-F) H&E staining (scale bar, 500 µm (E); scale bar, 200 µm (F)), (G) α-SMA staining (scale bar, 200 µm), **(H-I)** Masson staining (scale bar, 200 µm (H); scale bar, 200 µm (I)), **(J)** Oil Red O staining (scale bar, 500 µm), and **(K)** F4/80 staining (scale bar, 200 µm). Left panels, representative images. Right panels, quantification of stained area or a percentage of lesion area. “×” indicates necrotic area. **(L)** Plaque stability was significantly increased in SRC-3^-/-^ApoE^-/-^ mice. The data represent the mean ± SEM. The results are representative of three independent experiments. *P* values were calculated by unpaired two-tailed Student's t-test. *, *P*<0.05; ** *P*<0.01.

To determine whether SRC-3 is involved in the development of atherosclerosis, we measured SRC-3 expression in human atherosclerotic plaques. We collected atherosclerotic plaques and plaque-adjacent vasculature in the lower limb aorta and assayed SRC-3 by Western blotting. As shown in Figure 1A, SRC-3 protein expression in the atherosclerotic plaques in lower limb aortas was significantly higher than that in the plaque-adjacent vasculature. To further determine whether SRC-3 was increased in mouse models of atherosclerosis, whole aortas were isolated from 12-week chow-fed and WD-fed male ApoE^-/-^ mice for Western blotting. The results showed that SRC-3 protein expression was significantly increased in the aortas of WD-fed ApoE^-/-^ mice compared with chow-fed ApoE^-/-^ mice (Figure 1B). These results suggest that SRC-3 may be involved in atherosclerosis development.

To assess whether SRC-3 deficiency affects the development of atherosclerosis, SRC-3^-/-^ApoE^-/-^ mice were generated by crossing SRC-3^+/-^ mice with ApoE^-/-^ mice. Since a *LacZ* reporter was inserted into the 10th amino acid of SRC-3, which is controlled by the endogenous SRC-3 promoter in SRC-3^-/-^ApoE^-/-^ mice 15, we performed an X-gal staining assay to determine the pattern of SRC-3 expression in the aortic roots of SRC-3^-/-^ApoE^-/-^ mice. As shown in Figure 1C, SRC-3 expression (blue) was observed in endothelial cells (ECs) and vascular smooth muscle cells (VSMCs) in the aortic roots of chow-fed SRC-3^-/-^ApoE^-/-^ mice, and the signals in the aortic roots of WD-fed SRC-3^-/-^ApoE^-/-^ mice were stronger. After eight-week-old male SRC-3^-/-^ApoE^-/-^ and SRC-3^+/+^ApoE^-/-^ mice were fed a WD for 12 weeks, the Oil Red O-stained areas in the aortas of SRC-3^-/-^ApoE^-/-^ mice were reduced by 60% compared with those in SRC-3^+/+^ApoE^-/-^ mice (6.68% versus 2.43%; Figure 1D). Next, we analyzed the advanced atherosclerotic lesions in the aortic roots by H&E staining. Quantitative analysis of representative images showed that the lesion areas were much smaller in SRC-3^-/-^ApoE^-/-^ mice than in SRC-3^+/+^ApoE^-/-^ mice (Figure 1E). We also analyzed the necrotic cores of plaques, which are critical determinants of plaque vulnerability. Quantification of this index revealed a pronounced decrease in the total necrotic area in the lesions of SRC-3^-/-^ApoE^-/-^ mice (Figure 1F). Since body weight, plasma lipid levels, and glucose levels are associated with atherosclerosis development, we measured body weight, plasma lipid levels, and glucose levels in SRC-3^-/-^ApoE^-/-^ and SRC-3^+/+^ApoE^-/-^ mice after the mice were fed a WD for 12 weeks. Decreased plasma lipid levels and glucose levels were observed in SRC-3^-/-^ApoE^-/-^ mice after being fed a WD for 12 weeks (Supplementary Figure 1A). Collectively, these results suggest that SRC-3 deficiency attenuates the development of atherosclerosis.

To further examine the effect of SRC-3 deficiency on the stability of atherosclerotic plaques, we investigated the properties that affect plaque stability, including α-SMA content, collagen content, fibrous cap thickness, lipid accumulation, and macrophage numbers. The α-SMA content of aortic root lesions in SRC-3^-/-^ApoE^-/-^ mice was decreased compared with that in SRC-3^+/+^ApoE^-/-^ mice (Figure 1G). However, the collagen content of aortic root lesions was significantly increased in SRC-3^-/-^ApoE^-/-^ mice compared with SRC-3^+/+^ApoE^-/-^ mice (Figure 1H). Fibrous caps in the aortic root lesions of SRC-3^-/-^ApoE^-/-^ mice were thicker than those of SRC-3^+/+^ApoE^-/-^ mice (Figure 1I). Lipid staining with Oil Red O revealed a robust 36% decrease in lipid accumulation in the aortic root lesions of SRC-3^-/-^ApoE^-/-^ mice compared with SRC-3^+/+^ApoE^-/-^ mice (Figure 1J). Immunohistological staining of macrophages (F4/80-positive), which can form foam cells that are critical for atherogenesis, was performed, and there was greatly reduced staining of F4/80-positive cells in the aortic root lesions of SRC-3^-/-^ApoE^-/-^ mice compared with SRC-3^+/+^ApoE^-/-^ mice (Figure 1K). In summary, aortic plaque stability was significantly increased in SRC-3^-/-^ApoE^-/-^ mice compared with SRC-3^+/+^ApoE^-/-^ mice (Figure 1L). These results suggest that SRC-3 reduces the stability of atherosclerotic plaques in the aortic root.

### SRC-3 in endothelial cells promotes the development of atherosclerosis

**Figure 2:**
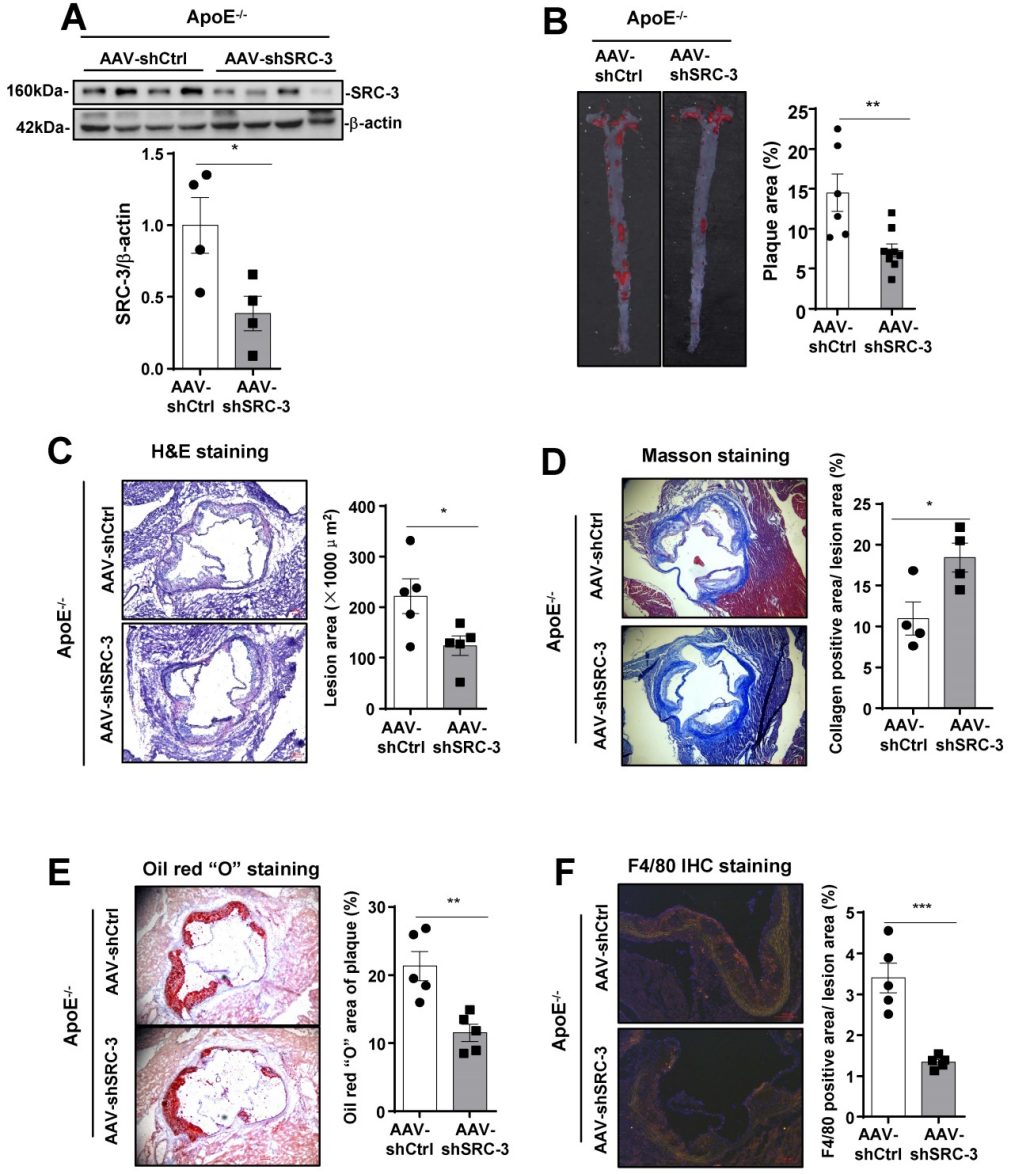
**SRC-3 in endothelial cells contributes to the development of atherosclerosis. (A)** Western blot showing that AAV-mediated SRC-3 shRNA decreased SRC-3 expression levels in the aortas of ApoE^-/-^ mice. **(B)** SRC-3 knockdown reduced WD-induced atherosclerotic plaque formation in ApoE^-/-^ mice. Representative images of *en face* Oil Red O-stained aortas from ApoE^-/-^ mice injected with AAV-mediated SRC-3 shRNA and scramble shRNA (left panel). Quantification of the plaque areas of aortas (right panel). The data represent the mean ± SEM. **(C-F)** Cross-sections of the aortic roots of ApoE^-/-^ mice injected with AAV-mediated SRC-3 shRNA and scramble shRNA were subjected to (C) H&E staining (scale bar, 100 µm), (D) Masson staining (scale bar, 500 µm), (E) Oil Red O staining (scale bar, 100 µm), **(F)** F4/80 staining (scale bar, 100 µm). Left panels, representative images. Right panels, quantification of stained area or a percentage of lesion area. The data represent the mean ± SEM. The results are representative of three independent experiments. *P* values were calculated by unpaired two-tailed Student's t-test. *, *P*<0.05; **, *P*<0.01; ***, *P*<0.001.

Our results demonstrated that SRC-3 was highly expressed in endothelial cells (Figure 1B). Therefore, we hypothesized that endothelial SRC-3 may play an important role in promoting the development of atherosclerosis. It has been reported that the transduction efficiency of the RGDLRVS-AAV9-cap plasmid in endothelial cells is significantly higher than both wild-type AAV2 and AAV9 *in vitro* 17, and the RGDLRVS-AAV9-cap plasmid has been used for endothelial-specific gene expression or knockdown in mouse models 18, 21. To downregulate SRC-3 expression in endothelial cells *in vivo*, we infected ApoE^-/-^ mice with endothelial-specific RGDKRVS-AAV9-mediated SRC-3 short hairpin RNA (shSRC-3) and negative control short hairpin RNA (shCtrl) by tail vein injection before feeding the mice a WD. The aortas of ApoE^-/-^ mice injected with AAV9-mediated shSRC-3 exhibited significantly reduced SRC-3 protein expression after the mice were fed a WD (Figure 2A). There were comparable plasma cholesterol levels and glucose levels between the AAV-shSRC-3 group and the AAV-shCtrl group after WD feeding (Supplementary Figure 1B). *En face* analysis of Oil Red O-stained atherosclerotic lesion area showed an approximate 2-fold decrease in the AAV-shSRC-3 group compared with the AAV-shCtrl group after WD feeding (Figure 2B). Staining of smooth muscle actin, collagen, lipids, and the macrophage marker F4/80 in the aortic roots in the AAV-shSRC-3 group and AAV-shCtrl group was performed to assess plaque composition. Consistent with the *en face* results, enhanced staining for collagen but reduced staining for lipids and the macrophage marker F4/80 were observed in AAV-shSRC-3 aortic roots (Figure 2C-F). These results demonstrate that SRC-3 in endothelial cells plays an important role in promoting the development of atherosclerosis.

### SRC-3 increases ICAM-1 expression during atherosclerosis development

**Figure 3:**
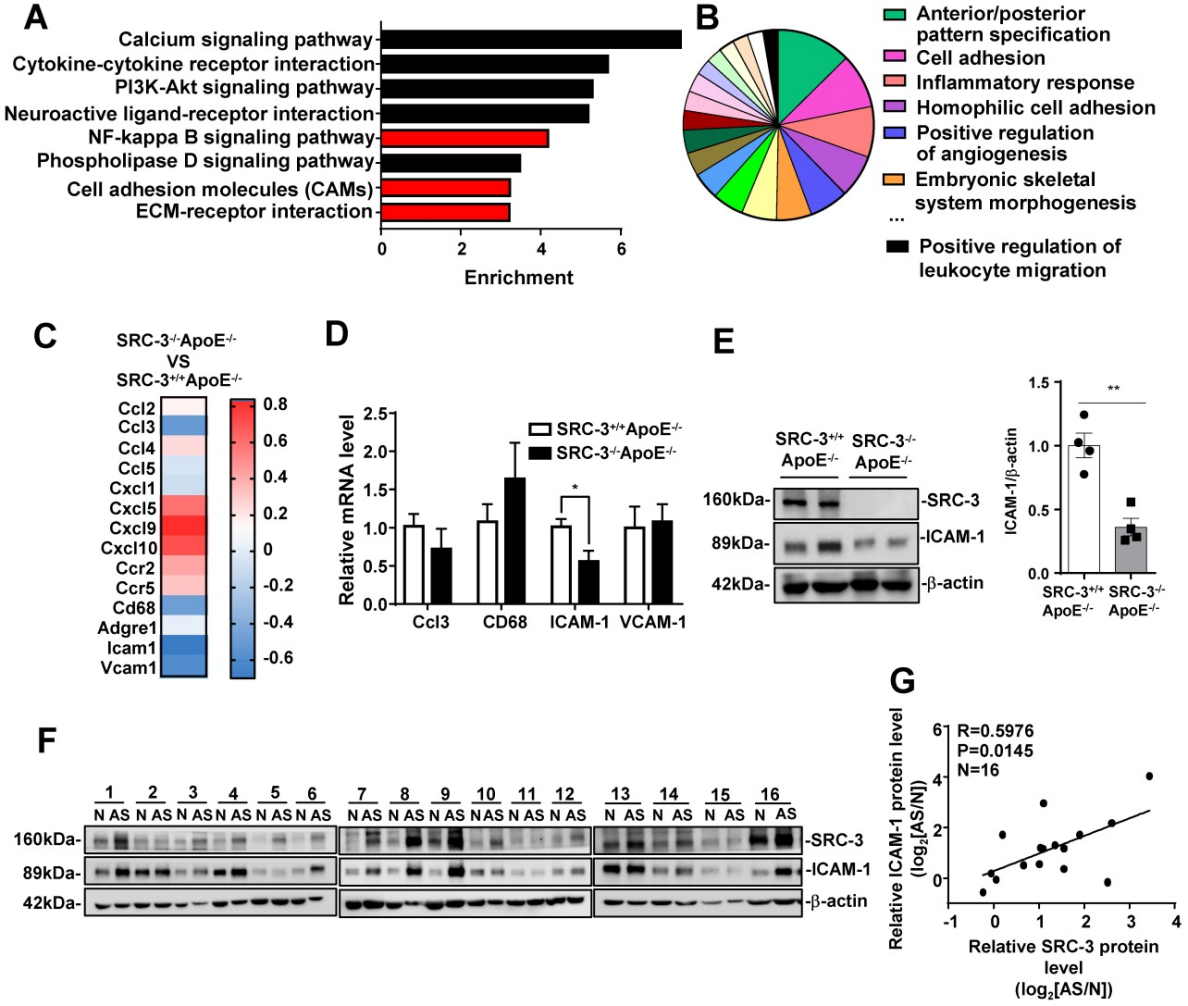
**SRC-3 increases ICAM-1 expression during atherosclerosis development. (A)** KEGG enrichment pathway analysis and **(B)** Gene Ontology (GO) biological process analysis of mRNA profiles in the aortas of SRC-3^+/+^ApoE^-/-^ and SRC-3^-/-^ApoE^-/-^ mice after WD feeding for 12 weeks. **(C)** Selected genes involved in leukocyte recruitment and proinflammatory markers are shown as a heat map. **(D)** The mRNA level of ICAM-1 in the aortas of SRC-3^-/-^ApoE^-/-^ mice was significantly decreased after WD feeding for 12 weeks. **(E)** The protein level of ICAM-1 in the aortas of SRC-3^+/+^ApoE^-/-^ and SRC-3^-/-^ApoE^-/-^ mice after WD feeding for 12 weeks. Each lane represents a pooled sample of three representative mice. **(F)** Western blot analysis of SRC-3 and ICAM-1 in 16 atherosclerotic plaques and plaque-adjacent vasculature in the lower limb aorta of accident patients. N represents plaque-adjacent vasculature in the lower limb aorta; AS represents atherosclerotic plaques in the lower limb aorta. **(G)** Correlation between SRC-3 and ICAM-1 protein levels in 16 atherosclerotic plaques and plaque-adjacent vasculature in the lower limb aorta. The data represent the mean ± SEM. The results are representative of three independent experiments. *P* values were calculated by unpaired two-tailed Student's t-test. *, *P*<0.05.

To examine the mechanism of SRC-3-mediated promotion of atherosclerosis, we performed messenger RNA (mRNA) sequencing in the aortas of WD-fed SRC-3^-/-^ApoE^-/-^ and SRC-3^+/+^ApoE^-/-^ mice. KEGG enrichment pathway analysis revealed eight enriched pathways (Figure 3A and E), including the NF-kappa B signaling pathway, cell adhesion molecules (CAMs), and ECM-receptor interaction. Gene Ontology (GO) analysis of enriched biological processes indicated that SRC-3 was associated with cell adhesion and the inflammatory response (Figure 3B). Because the aortas of WD-fed SRC-3^-/-^ApoE^-/-^ mice had reduced macrophage recruitment (Figure 1K), we focused on the expression of cell adhesion molecules, CC and CXC chemokines and their receptors, which are responsible for leukocyte recruitment (Figure 3C). The RNA-Seq results revealed decreased expression of CCL3, CD68, ICAM-1, and VCAM-1 in the aortas of WD-fed SRC-3^-/-^ApoE^-/-^ mice (Figure 3C); however, real-time quantitative PCR showed that only ICAM-1 expression was significantly reduced in the aortas of WD-fed SRC-3^-/-^ApoE^-/-^ mice (Figure 3D). ICAM-1 is involved in the progression of atherosclerosis in ApoE^-/-^ mice 22, 23, 24, 25, 26. Therefore, ICAM-1 was selected for further investigation. Western blot analysis showed that the protein levels of ICAM-1 were significantly decreased in the aortas of WD-fed SRC-3^-/-^ApoE^-/-^ mice compared with SRC-3^+/+^ApoE^-/-^ mice (Figure 3E), suggesting that SRC-3 upregulates ICAM-1 expression. Consistently, the protein levels of SRC-3 were positively correlated with the protein levels of ICAM-1 in human atherosclerotic lesions (Figure 3E and F). Taken together, these results suggest that SRC-3 promotes ICAM-1 expression during atherosclerosis development.

### SRC-3 promotes ICAM-1 expression by enhancing NF-κB signaling

**Figure 4:**
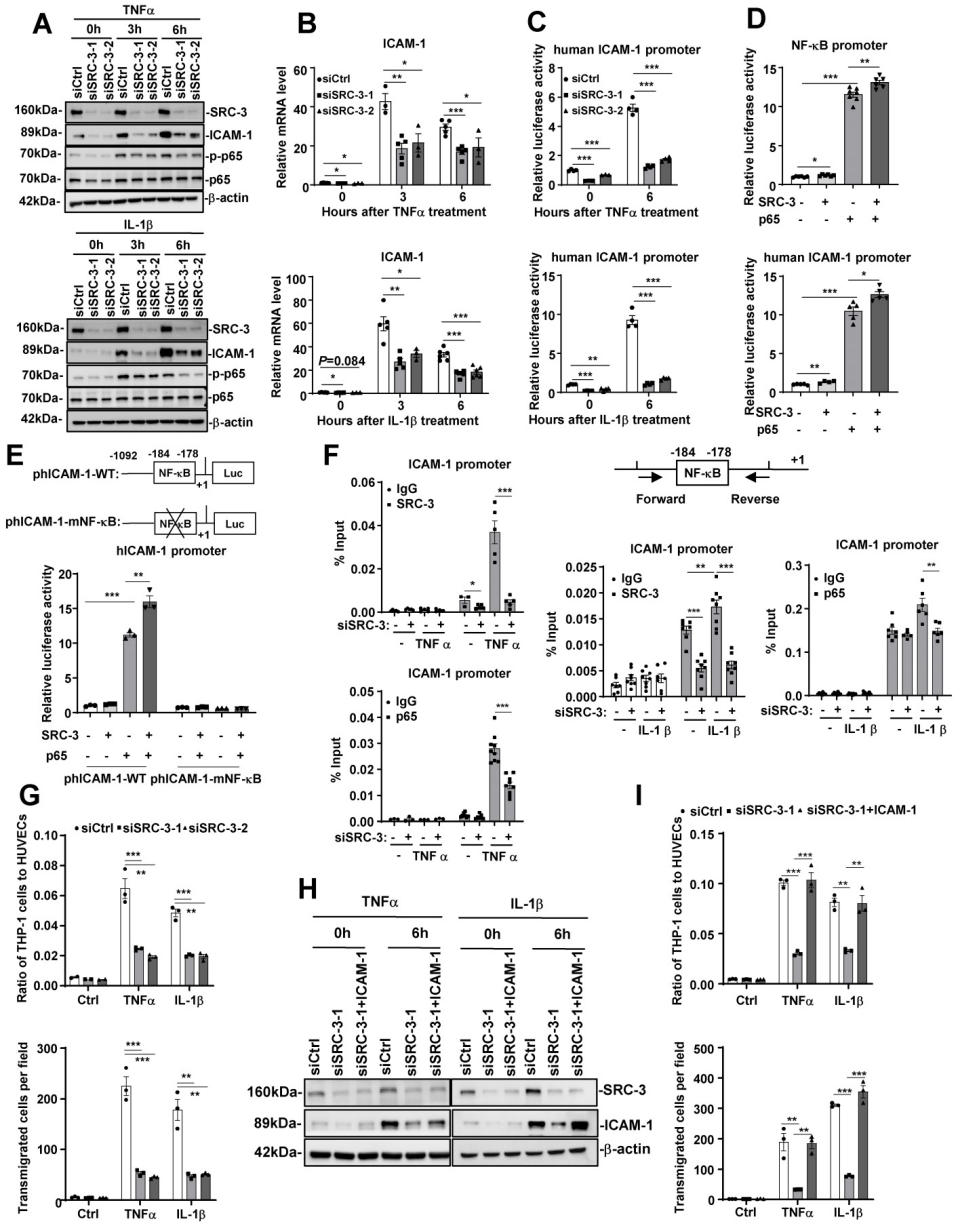
**SRC-3 regulates ICAM-1 expression via enhancing NF-κB signaling. (A)** The protein levels of SRC-3 and ICAM-1 in SRC-3 siRNA-transfected HUVECs were significantly reduced compared with those in scrambled siRNA-transfected HUVECs after TNFα or IL-1β treatment. **(B)** The mRNA level of ICAM-1 in the SRC-3 siRNA-transfected HUVECs was markedly decreased after TNFα or IL-1β treatment. **(C)** ICAM-1 promoter activity was reduced in SRC-3 siRNA-transfected HUVECs after TNFα or IL-1β treatment. **(D)** SRC-3 cooperated with p65 to enhance the activity of the NF-κB reporter (upper panel) and ICAM-1 promoter (lower panel). **(E)** NF-κB binding site mutation abolished NF-κB-mediated ICAM-1 promoter activity. **(F)** The recruitment of SRC-3 and p65 was significantly reduced in SRC-3 siRNA-transfected HUVECs after TNFα or IL-1β treatment (right panel). Position of the subfragments detected by ChIP assays (left panel). **(G)** The SRC-3 siRNA-transfected HUVECs monolayer exhibited a significantly decreased number of adhered (upper panel) and transmigrated (lower panel) THP-1 cells after TNFα or IL-1β treatment. **(H)** Western blot showing ICAM-1 overexpression in SRC-3 siRNA-transfected HUVECs. **(I)** ICAM-1 overexpression in SRC-3 siRNA-transfected HUVECs rescued monocyte attachment to HUVECs and monocyte transendothelial migration after TNFα or IL-1β treatment. The data represent the mean ± SEM of three independent experiments. *P* values were calculated by unpaired two-tailed Student's t-test. *, *P*<0.05; **, *P*<0.01;***, *P*<0.001.

Since both the mRNA and protein levels of ICAM-1 were significantly decreased in the aortas of WD-fed SRC-3^-/-^ApoE^-/-^ mice compared with SRC-3^+/+^ApoE^-/-^ mice, SRC-3 may regulate ICAM-1 expression at the transcriptional level. To determine whether SRC-3 can regulate ICAM-1 expression in endothelial cells, we transfected SRC-3-specific small interfering RNA (siSRC-3) into human umbilical vein endothelial cells (HUVECs) to knock down SRC-3 and examined ICAM-1 expression. As shown in Figure 4A and B, the protein and mRNA levels of ICAM-1 in siSRC-3-transfected HUVECs were significantly reduced compared with scrambled siRNA (siCtrl)-transfected HUVECs before and after TNFα or IL-1β treatment. Restoration of SRC-3 expression in SRC-3-knockdown HUVECs could rescue ICAM-1 expression after TNFα or IL-1β treatment (Supplementary Figure 2A and B). Furthermore, we examined whether SRC-3 could promote ICAM-1 transcription by transfecting the ICAM-1 promoter-reporter plasmid into wild-type and SRC-3-knockdown HUVECs. As shown in Figure 4C, TNFα or IL-1β treatment markedly induced ICAM-1 promoter activity in scramble HUVECs, whereas SRC-3 knockdown significantly reduced the induction of ICAM-1 promoter activity. These results suggest that SRC-3 promotes ICAM-1 expression at the transcriptional level.

It has been reported that ICAM-1 expression can be induced by NF-κB 27. Because TNFα or IL-1β treatment can induce NF-κB activation (Figure 4A) and SRC-3 can interact with p65 to enhance p65-mediated NF-κB reporter activation (Figure 4D, upper panel), we hypothesized that SRC-3 may enhance NF-κB-mediated ICAM-1 expression. We tested this hypothesis by transfecting HUVECs with an ICAM-1 promoter-reporter plasmid with SRC-3, p65, or both SRC-3 plus p65 expression plasmids. Transfection of p65 or SRC-3 alone induced a 10-fold or 1.3-fold increase in ICAM-1 promoter activity (Figure 4D, lower panel), respectively, whereas cotransfection of p65 and SRC-3 induced a 13-fold increase in ICAM-1 promoter activity (Figure 4D, lower panel). These results suggest that SRC-3 can enhance NF-κB-mediated ICAM-1 expression. To further confirm the role of p65 in NF-κB-mediated activation of the ICAM-1 promoter, we mutated the NF-κB binding site on the ICAM-1 promoter. As shown in Figure 4E, cotransfection of p65 and SRC-3 induced the activity of the wild-type ICAM-1 promoter by approximately 15-fold, while mutating the NF-κB binding site dramatically abolished NF-κB-mediated ICAM-1 promoter activity, suggesting that the NF-κB binding site is essential for NF-κB-mediated activation of the ICAM-1 promoter. We next determined whether SRC-3 and p65 could be recruited to the NF-κB binding site on the promoter after TNFα or IL-1β treatment. As shown in Figure *4F*, SRC-3 and p65 bound to the NF-κB binding site on the ICAM-1 promoter after TNFα or IL-1β treatment, whereas SRC-3 knockdown in HUVECs markedly reduced the recruitment of p65 and SRC-3 to the ICAM-1 promoter, suggesting that SRC-3 and p65 are recruited to the NF-κB binding site to enhance NF-κB-mediated activation of the ICAM-1 promoter after TNFα or IL-1β treatment.

In response to endothelial cell activation, various leukocytes are recruited, which is a basic event in vascular inflammation. The processes that participate in inflammatory cell recruitment include leukocyte-endothelial adhesion and transmigration. To determine the role of endothelial SRC-3 in vascular inflammation, we performed proinflammatory cytokine-mediated monocyte-endothelial cell adhesion and transmigration assays. As shown in Figure 4G, the siSRC-3-transfected HUVEC monolayer exhibited a marked decrease in the number of adhered and transmigrated THP-1 cells compared with the siCtrl-transfected HUVEC monolayer after TNFα or IL-1β treatment. We next determined whether the restoration of ICAM-1 expression in SRC-3-knockdown HUVECs could rescue the number of adhered and transmigrated THP-1 cells after TNFα or IL-1β treatment. As shown in Figure 4H and I, ectopic expression of ICAM-1 in SRC-3-knockdown HUVECs rescued the number of adhered and transmigrated THP-1 cells after TNFα or IL-1β treatment. These results suggest that SRC-3 promotes the adhesion and transmigration of THP-1 cells at least in part by increasing ICAM-1 expression.

### ICAM-1 expressed in endothelial cells is required for atherosclerosis development

**Figure 5:**
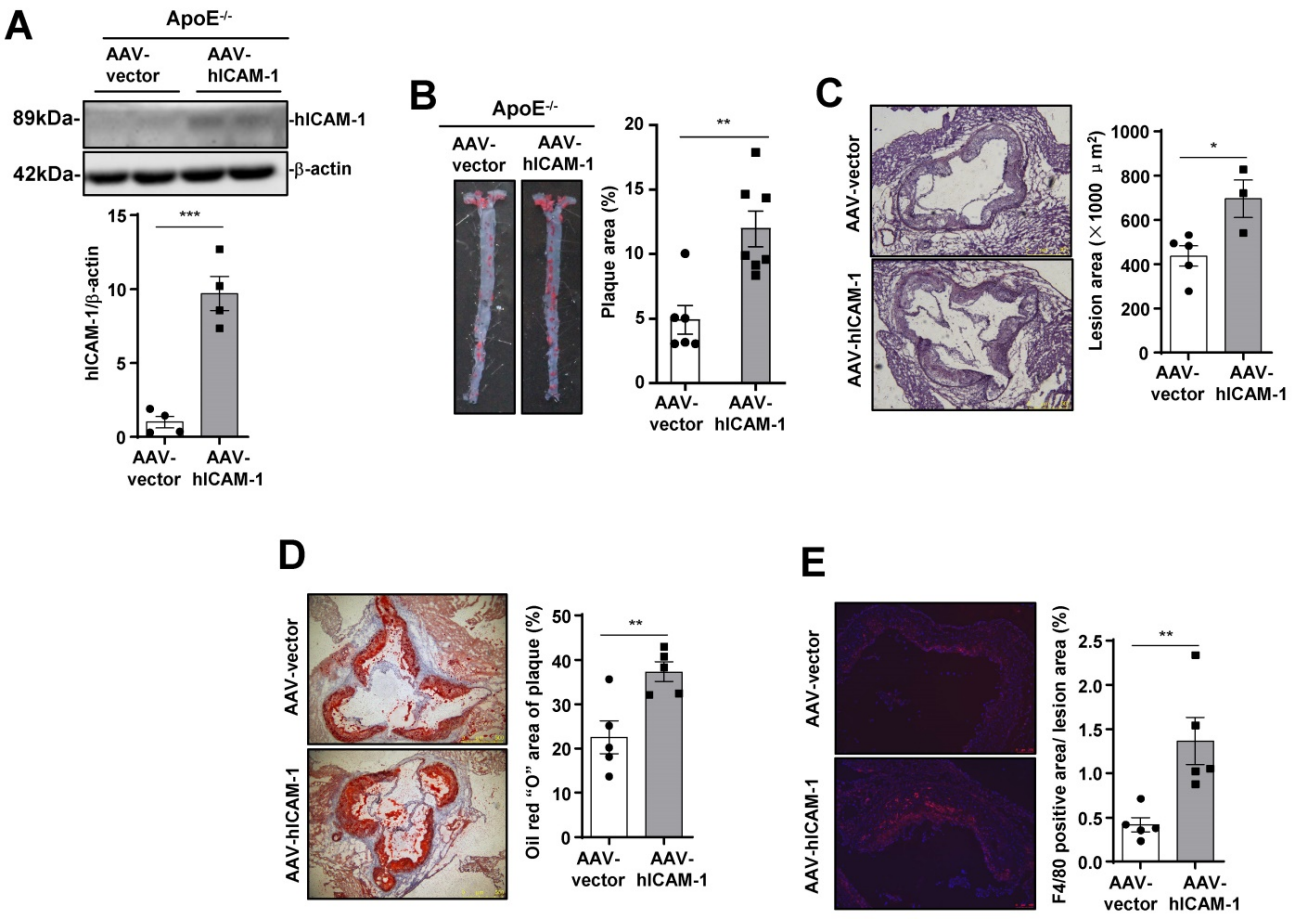
**ICAM-1 in endothelial cells is required for atherosclerosis development. (A)** Western blot analysis showing increased hICAM-1 expression in the aortas of mice injected with AAV expressing hICAM-1. **(B)** hICAM-1 overexpression accelerated WD-induced atherosclerotic plaque formation in ApoE^-/-^ mice. Representative images of *en face* Oil Red O-stained aortas from ApoE^-/-^ mice injected with AAV expressing hICAM-1 and vector (left panel). Quantification of the plaque areas of aortas (right panel). The data represent the mean ± SEM. **(C-E)** Cross-sections of the aortic roots from ApoE^-/-^ mice injected with AAV-hICAM-1 and vector were subjected to **(C)** H&E staining (scale bar, 100 µm), **(D)** Oil Red O staining (scale bar, 100 µm), and (E) F4/80 staining (scale bar, 100 µm). Left panels, representative images. Right panels, quantification of stained area or a percentage of lesion area. The data represent the mean ± SEM. The results are representative of three independent experiments. *P* values were calculated by unpaired two-tailed Student's t-test. *, *P*<0.05; **, *P*<0.01;***, *P*<0.001.

To verify that ICAM-1 in endothelial cells promotes atherosclerotic plaque formation in mice, we infected ApoE^-/-^ mice with endothelial-specific RGDKRVS-AAV9-ICAM-1 or scramble virus by tail vein injection before feeding the mice a WD. Mice that were injected with endothelial-specific AAV9-ICAM-1 virus showed significantly higher ICAM-1 expression (Figure 5A) and markedly increased plaque formation (Figure 5B) and lesion area (Figure 5C) than mice that were injected with the scramble control virus. Furthermore, staining for lipids and the macrophage marker F4/80 in the aortic roots in the AAV9-scramble control group and AAV9-ICAM-1 expression group was performed to assess plaque composition. Consistent with the *en face* results, increased staining for lipids and the macrophage marker F4/80 was observed in AAV9-hICAM-1-infected aortic roots (Figure 5C and E). There was no difference in plasma lipid content levels and plasma glucose levels between the AAV9-scramble and AAV9-hICAM-1 groups (Supplementary Figure 3A). Conversely, we knocked down mouse ICAM-1 by using an endothelial-specific AAV9-shICAM-1 virus. Plasma lipid levels and plasma glucose levels were comparable between the AAV-scramble control and AAV-shICAM-1 virus-infected groups (Supplementary Figure 3B). Mice that were injected with the endothelial-specific AAV9-shICAM-1 virus exhibited significantly decreased atherosclerotic plaque formation and lesion areas (Supplementary Figure 4A and B). Consistent with the *en face* results, reduced staining for lipids and the macrophage marker F4/80 was observed in AAV9-shICAM-1 virus-infected aortic roots (Supplementary Figure 4C-E). These results demonstrate that ICAM-1 in endothelial cells is required for the development of atherosclerosis.

### Pharmacological inhibition of SRC-3 reduces atherosclerosis

**Figure 6:**
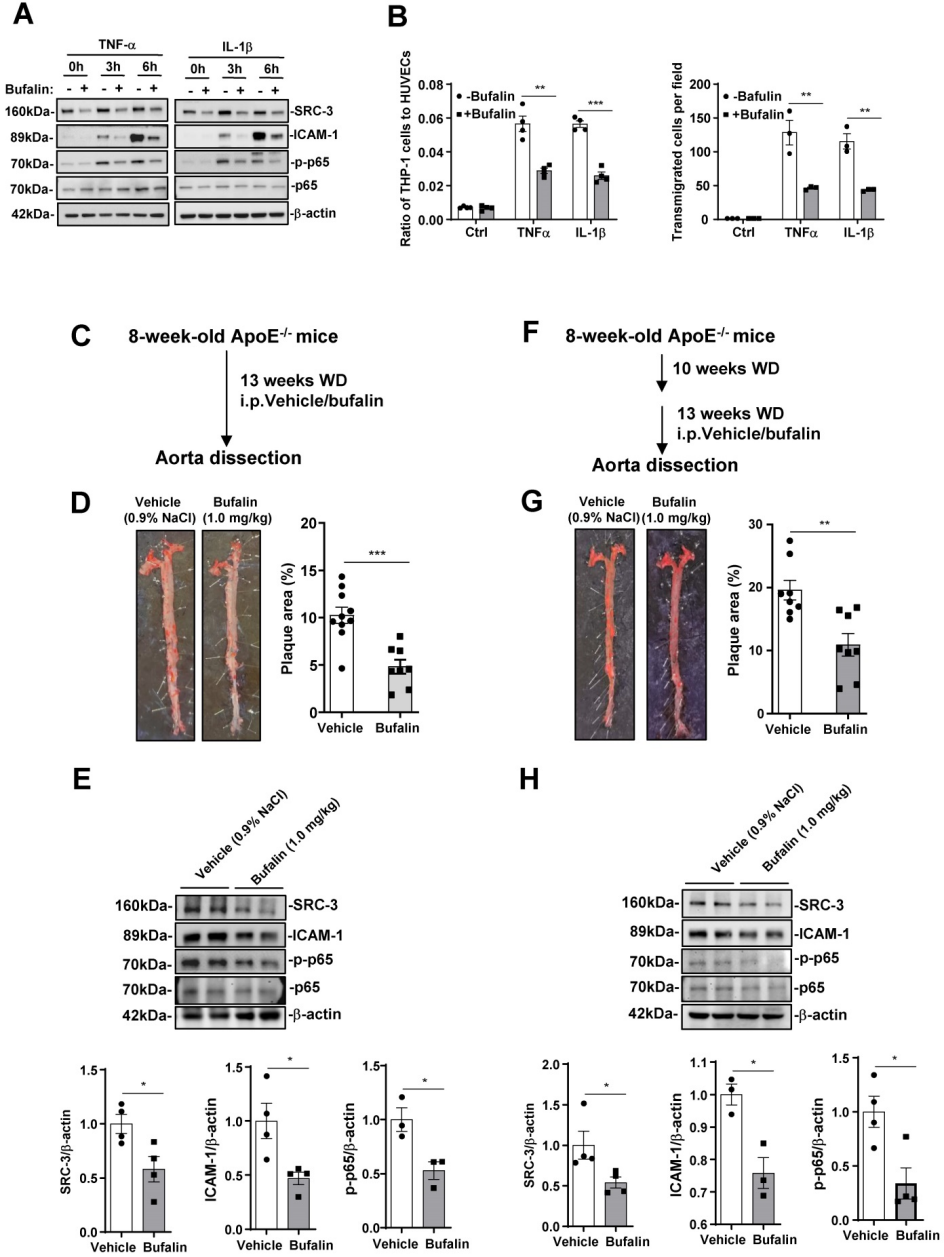
** Pharmacological inhibition of SRC-3 reduces atherosclerosis. (A)** The protein levels of SRC-3, ICAM-1 and p-p65 in bufalin-treated HUVECs were significantly reduced compared with those in vehicle-treated HUVECs after TNFα or IL-1β treatment. The bufalin-treated HUVECs monolayer resulted in a dramatically decreased number of adhered (upper panel) and transmigrated (lower panel) THP-1 cells after TNFα or IL-1β treatment. **(B-C)** ApoE^-/-^ mice were administered vehicle or bufalin (1.0 mg/kg, six times a week) by intraperitoneal injection for 13 weeks concomitant with WD feeding. (B) Dosing regimen (ApoE^-/-^ prevention model). (C) Representative images of *en face* Oil Red O-stained aortas from ApoE^-/-^ mice treated with vehicle or bufalin (left panel). Quantification of the plaque areas of aortas (right panel). The data represent the mean ± SEM. **(D)** The protein levels of SRC-3 and ICAM-1 in the aortas of ApoE^-/-^ mice treated with bufalin were significantly reduced in the ApoE^-/-^ prevention model. **(E-F)** ApoE^-/-^ mice were fed a WD for 10 weeks and then treated with vehicle or bufalin (1.0 mg/kg, six times a week) by intraperitoneal injection for 13 weeks concomitant with WD feeding. (E) Dosing regimen (ApoE^-/-^ regression model). (F) Representative images of en face Oil Red O-stained aortas from ApoE^-/-^ mice treated with vehicle or bufalin (left panel). Quantification of the plaque areas of aortas (right panel). The data represent the mean ± SEM. **(G)** The protein levels of SRC-3 and ICAM-1 in the aortas of ApoE^-/-^ mice treated with bufalin were significantly reduced in the ApoE^-/-^ regression model. The data represent the mean ± SEM. The results are representative of three independent experiments. *P* values were calculated by unpaired two-tailed Student's t-test. *, *P*<0.05; **, *P*<0.01;***, *P*<0.001.

Since SRC-3 deficiency prevents atherosclerosis development, we assessed the protective effect of bufalin, a small-molecule inhibitor of SRC-3 that has been used in tumor therapy 28, 29, in the development of atherosclerosis. We first performed proinflammatory cytokine-mediated monocyte-endothelial cell adhesion and transmigration assays to determine whether bufalin alleviates macrophage recruitment *in vitro*. As shown in Figure 6A, the expression of SRC-3 and ICAM-1 in HUVECs was significantly reduced by bufalin treatment. Additionally, bufalin treatment significantly decreased the expression of p-p65 (Figure 6A). Consequently, a significant reduction in adhered and transmigrated THP cells was observed in the presence of bufalin-treated HUVECs in response to TNFα or IL-1β treatment (Figure 6B).

We then determined the protective effect of bufalin on atherosclerosis by using ApoE^-/-^ prevention model. Bufalin was administered to ApoE^-/-^ mice (1.0 mg/kg, six times a week) by intraperitoneal injection for 13 weeks during WD feeding (Figure 6C). Bufalin-treated mice exhibited decreased atherosclerotic plaque formation compared with those treated with vehicle only (Figure 6D). Furthermore, the protein levels of SRC-3 and ICAM-1 in the aortas from ApoE^-/-^ mice treated with bufalin were also significantly reduced after the mice were fed a WD for 13 weeks (Figure 6E). Reduced p-p65 was observed in the aortas of mice treated with bufalin after WD feeding for 13 weeks (Figure 6F). Bufalin treatment did not significantly affect plasma lipid or glucose levels (Supplementary Figure 5A).

It is well known that atherosclerosis is an advanced disease, and so we performed a regression model by treating ApoE^-/-^ mice that were fed WD alone for 10 weeks with bufalin for 13 weeks during WD feeding (Figure 6F). Bufalin treatment further diminished plaque development (Figure 6G). The levels of SRC-3, ICAM-1, and p-p65 in the aortas of bufalin-treated mice were also reduced compared with those of vehicle-treated mice (Figure 6H). Bufalin treatment significantly increased TC and HDL-C, but decreased glucose levels (Supplementary Figure 5B). These results demonstrate that the pharmacological SRC-3 inhibitor bufalin can ameliorate the development of atherosclerosis.

## Discussion

**Figure 7:**
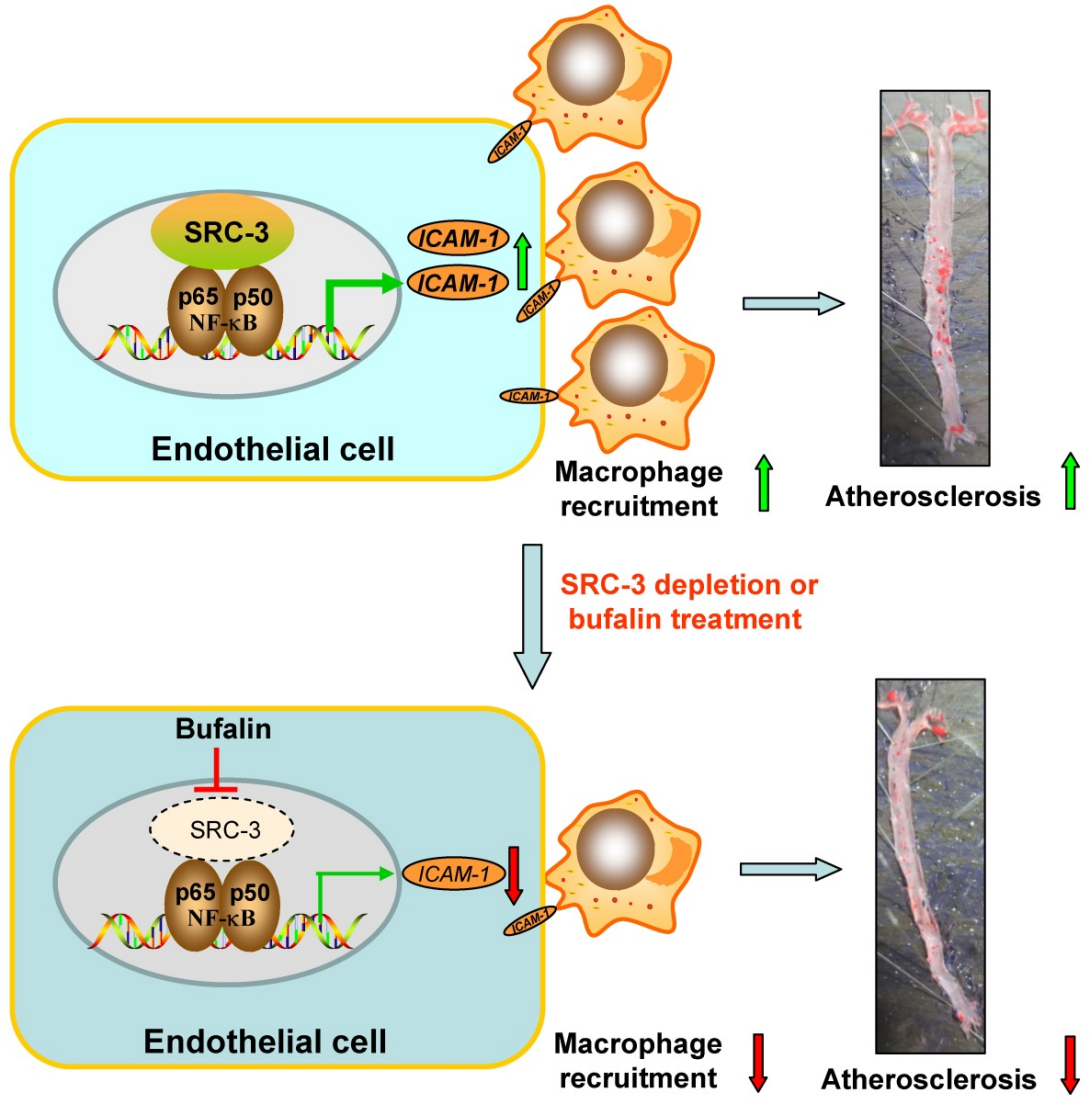
Schematic model of the mechanism by which SRC-3 accelerates atherosclerosis development. SRC-3 promotes atherosclerosis development by increasing ICAM-1 transcription by enhancing the function of NF-κB in endothelial cells to promote macrophage recruitment. SRC-3 depletion or pharmacological inhibition of SRC-3 by bufalin ameliorates atherosclerosis development through decreasing endothelial ICAM-1 expression and macrophage recruitment via reduction of NF-κB function.

Lipid-mediated inflammation of the vessel wall is a key initiating event in atherosclerosis that activates endothelial cells to recruit macrophages in a leukocyte adhesion molecule-dependent manner 30. In this study, we found that global SRC-3 deficiency or endothelial SRC-3 knockdown on an ApoE^-/-^ background ameliorated the development of atherosclerosis, suggesting that SRC-3 could promote atherosclerosis development. Mechanistically, SRC-3 promoted atherosclerosis development by increasing ICAM-1 expression at the transcriptional level by enhancement of NF-κB function in endothelial cells to promote macrophage recruitment (Figure 7). SRC-3 depletion or pharmacological inhibition of SRC-3 by bufalin ameliorated atherosclerosis development, at least in part by decreasing endothelial ICAM-1 expression via reduction of NF-κB function (Figure 7).

In this study, we showed that the genetic ablation of SRC-3 prevented the development of atherosclerosis in ApoE^-/-^ mice, indicating that SRC-3 injures the vasculature during atherosclerosis. A previous study showed that SRC-3 was required for estrogen-induced inhibition of neointima formation during vascular trauma, which may be caused by suppressing of vascular cell proliferation 13. Recently, it has been reported that SRC-1/3 deficiency can cause noncompaction cardiomyopathy-like abnormalities in the hearts of newborn and adult mice, which are caused by the inhibition of cardiomyocyte proliferation and differentiation 14, suggesting that SRC-3 has a vasoprotective role. These studies demonstrate that SRC-3 has injures or protects the vasculature in different cardiovascular diseases through diverse mechanisms.

Advanced atherosclerotic lesions, which are essentially a nonresolving inflammatory condition, can cause the formation of the vulnerable plaques, increasing the risk of plaque rupture. Vulnerable plaques are characterized by the formation of necrotic cores and thinning of the fibrous cap. The necrotic core is the result of increased macrophage death and impaired efferocytosis 8. H&E staining of the aortic roots showed that the total necrotic areas in the aortic root lesions of SRC^-/-^ApoE^-/-^ mice were smaller than those in SRC-3^+/+^ApoE^-/-^ mice, and fibrous caps in the aortic root lesions of SRC-3^-/-^ApoE^-/-^ mice were thicker than those of SRC-3^+/+^ApoE^-/-^ mice. In human atherosclerotic-associated cardiovascular events, plaque morphology is a more critical predictor of plaque rupture than plaque size, and the size of the necrotic core plays a primary role in plaque vulnerability 16. Other features, such as high levels of inflammatory cytokines and significant accumulation of lipids also contribute to the vulnerability of atherosclerotic plaques 31. In this study, analysis of atherosclerotic plaques revealed that SRC-3^-/-^ApoE^-/-^ mice exhibited notably decreased infiltration of macrophages, decreases in α-SMA levels and lipid accumulation and an increase in collagen content. Advanced necrotic cores and other deteriorated features of vulnerable plaques are responsible for the majority of clinical events.

Our results showed that global SRC-3 deficiency on the ApoE^-/-^ background ameliorated the development of atherosclerosis. Previous studies have shown that RGDLRVS-AAV9-cap plasmid-based virus preferentially mediates genes expression in endothelial cells *in vitro* and *in vivo* 17, 18, 21. Therefore, ApoE^-/-^ mice were infected with endothelial-specific RGDKRVS-AAV9-shSRC-3 or control shRNA virus by tail vein injection before being fed a WD, and a model of atherosclerosis was constructed. We found that mice on the ApoE^-/-^ background that were infected with endothelial-specific AAV9-shSRC-3 also exhibited significant reductions in atherosclerotic plaques, similar to the effects of global SRC-3 deficiency on the ApoE^-/-^ background, suggesting that endothelial SRC-3 contributes to atherosclerosis development. Additionally, SRC-3^-/-^ApoE^-/-^ mice exhibited markedly decreased levels of plasma lipids and glucose compared with SRC-3^+/+^ApoE^-/-^ mice after being fed a WD for 12 weeks. However, mice on the ApoE^-/-^ background that were infected with endothelial-specific AAV9-shSRC-3 virus did not exhibit significantly altered levels of plasma lipids or glucose. Therefore, the decrease in plasma lipid and glucose levels due to global SRC-3 deficiency on the ApoE^-/-^ background could contribute to the reduction in atherosclerotic plaque formation in a manner that is independent of the endothelial cells.

ICAM-1 belongs to the Ig superfamily and is composed of a transmembrane domain and a short cytoplasmic domain 32. This factor is expressed by endothelial cells, epithelial cells, all leukocyte subsets 33, platelets 34, and vascular smooth muscle cells 35. ICAM-1 can mediate leukocyte rolling and firm adhesion to the vessel wall and leukocyte transmigration across the endothelial layer 36, promoting vascular inflammation. It has been reported that ICAM-1 deficiency or inhibition protects against the development of atherosclerosis in ApoE^-/-^ mice 22, 23, 24, 25, 26. In this study, we found that ICAM-1 overexpression in the vascular endothelial cells of mice infected with AAV9-hICAM-1 accelerated atherosclerotic plaque formation after the mice were fed a WD for 12 weeks, whereas ICAM-1 knockdown in the vascular endothelial cells of mice infected with AAV9-shICAM-1 virus alleviated atherosclerotic plaque formation after being fed WD for 12 weeks. Furthermore, ectopic ICAM-1 expression in SRC-3-knockdown HUVECs increased the number of adhered and transmigrated THP-1 cells compared with that in SRC-3-knockdown HUVECs. These results suggest that endothelial ICAM-1 plays an import role in promoting the development of atherosclerosis, at least in part through increasing leukocyte adhesion and transmigration and is sufficient to promote atherosclerosis development.

Bufalin is a major bioactive monomer of the traditional Chinese medicine Chan Su, which is acquired from the skin and parotid venom glands of the Chinese toad 37, 38. It has been reported that bufalin is a small-molecule inhibitor of SRC-3 that can directly bind to the receptor interacting domain of the SRC-3 protein to promote its degradation 28. In this study, bufalin treatment of ApoE^-/-^ mice ameliorated atherosclerotic plaque formation in an ApoE^-/-^ prevention model and regression model, suggesting that bufalin plays an important protective role against atherosclerosis. Furthermore, the protein levels of SRC-3 and ICAM-1 were reduced in the aortas of bufalin-treated ApoE^-/-^ mice after the mice were fed a WD for 13 weeks. We found that bufalin suppressed the protein expression of p-p65 and p65 in the aortas of bufalin-treated ApoE^-/-^ mice after the mice were fed a WD for 13 weeks. Additionally, bufalin-treated HUVECs exhibited significant reductions in adhered and transmigrated monocytes in response to TNFα or IL-1β, and bufalin treatment reduced the protein levels of SRC-3, ICAM-1, and p-p65 in HUVECs, similar to the effect on bufalin-treated ApoE^-/-^ mice, suggesting that endothelial cells are targets of bufalin. These results demonstrate that SRC-3 pathway inhibited by bufalin was involved in atherosclerosis development, at least in part through reducing ICAM-1 expression via reducing NF-κB signaling. Recent studies have shown that bufalin exerts anticancer activity on various cancers 29. Furthermore, Chan Su has been widely used in the clinical treatment of malignancies in China and exhibits good therapeutic efficacy 39, 40. Thus, the SRC-3 inhibitor bufalin may not only represent a new drug for the prevention and treatment of atherosclerosis, but may also be safe for SRC-3-targeted cancer therapies at the experimental and clinical levels.

NF-κB can regulate endothelial ICAM-1 expression at the transcriptional level 41, 42. Furthermore, endothelial NF-κB deficiency decreased the expression of leukocyte adhesion molecules, attenuated macrophage recruitment to atherosclerotic plaques and decreased the expression of proinflammatory cytokines in the aorta 43, 44, suggesting that endothelial NF-κB is required for promoting atherosclerotic plaque formation. The present study showed that NF-κB signaling was inhibited in aortas of SRC-3^-/-^ApoE^-/-^ mice after WD feeding for 12 weeks or SRC-3-knockdown HUVECs after TNFα and IL-1β treatment, suggesting that SRC-3 can activate NF-κB. Additionally, it has been reported that SRC-3 can serve as a coactivator for p65 to enhance its transcriptional activity 12, 45, 46. Our results showed that SRC-3 could be recruited to the p65 binding site on the ICAM-1 reporter/promoter and that SRC-3 could cooperate with p65 to enhance ICAM-1 transcription, suggesting that SRC-3 coactivates NF-κB to upregulate ICAM-1 transcription. Although we found a consistent transcription factor AP-1 binding site (TGACTCGCA) at the ICAM-1 promoter, our results showed that SRC-3 could not cooperate with AP-1 to induce ICAM-1 promoter activity (Supplementary Figure 6). Certainly, we could not exclude that SRC-3 could coactivate other key transcription factors in the endothelial cells to affect ICAM-1 expression.

Given that macrophage foam cell formation is critical for the atherosclerosis development, we investigated the effect of SRC-3 on macrophage foam cell formation demonstrated by affecting oxLDL uptake. As shown in Supplementary Figure 7B and C, oxLDL uptake was significantly reduced in two SRC-3-knockdown stable RAW264.7 cell lines compared with control RAW264.7 cell line (shCtrl), suggesting that SRC-3 knockdown reduces macrophage foam cell formation. To determine whether bufalin treatment affects SRC-3 function in macrophages, we first assessed the effect of bufalin treatment on SRC-3 expression in macrophages. As shown in Supplementary Figure 7D, bufalin treatment didn't decreased SRC-3 expression in macrophages, whereas bufalin treatment could significantly reduced SRC-3 expression in HUVECs. It suggests that the effect of bufalin on SRC-3 expression may be cell context-dependent.

In summary, our study shows that endothelial SRC-3 deficiency or pharmacological inhibition prevents atherosclerosis development by decreasing endothelial ICAM-1 expression, resulting in the inhibition of macrophage recruitment. Thus, SRC-3 is a potential target for atherosclerosis prevention and therapy.

## Supplementary Material

Supplementary figures and table.

## Abbreviations

SRC-3: Steroid receptor coactivator 3
WD: Western diet
AAV: Adeno-associated virus
ICAM-1: Intercellular cell adhesion molecule-1
VCAM-1: Vascualr cell adhesion molecular-1
MCP-1: Monocyte chemoattractant protein-1
HUVEC: Human umbilical vein endothelial cell
EC: Endothelial cell
TNFα: Tumor necrosis factor α
IL-1β: Interleukin 1β
shCtrl: Negative control short hairpin RNA
shSRC-3: SRC-3-specific short hairpin RNA
siCtrl: Scrambled small interfering RNA
siSRC-3: SRC-3-specific small interfering RNA
H&E: Haematoxylin and eosin
