# Supplementary material for: SRC-3 deficiency prevents atherosclerosis development by decreasing endothelial ICAM-1 expression to attenuate macrophage recruitment
Source: Int J Biol Sci. 2022 Oct 3;18(15):5978–93. doi: 10.7150/ijbs.74864 (PMC9576506; doi:10.7150/ijbs.74864)
Supplement: Supplementary file 1 — Supplementary figures and table. [file ijbsv18p5978s1.pdf]

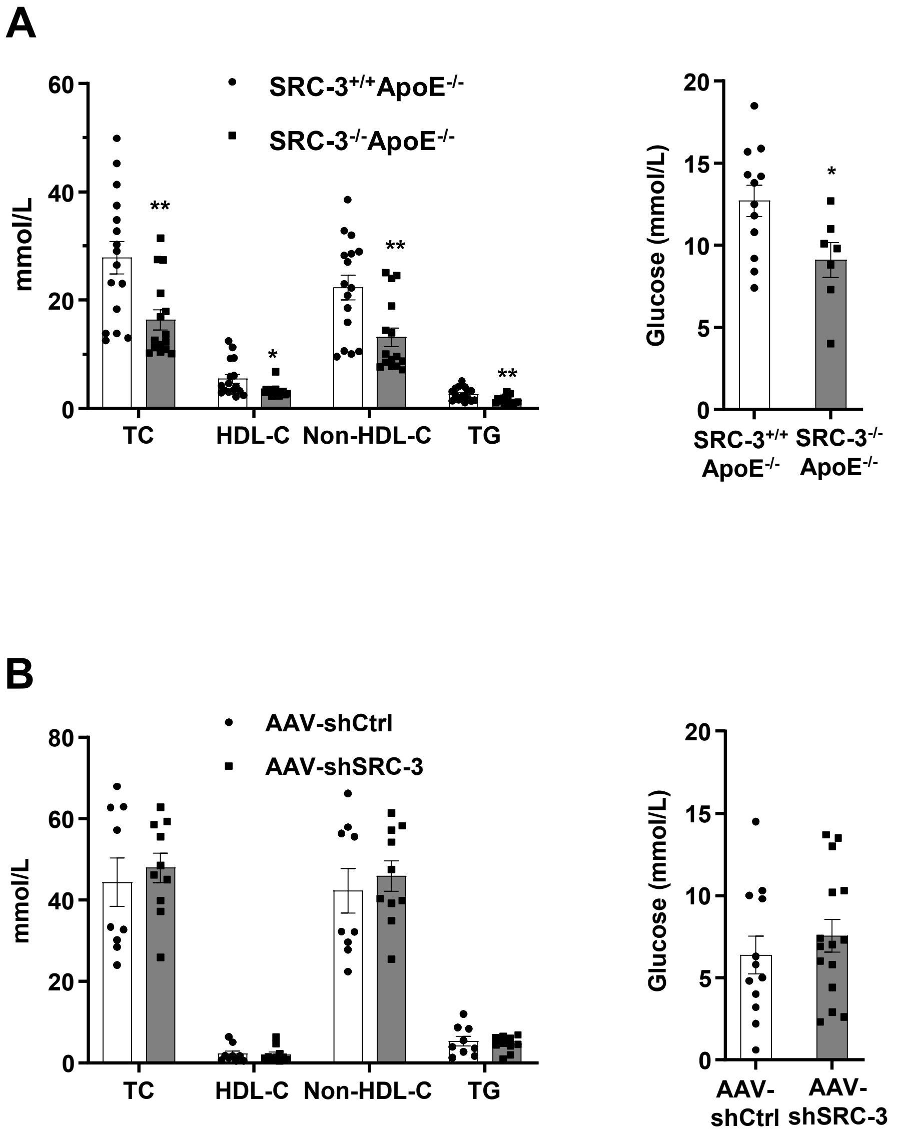

Supplementary Figure 1. Lipid levels and plasma glucose in SRC-3$^{-/-}$ ApoE$^{-/-}$ (A) or AAV-shSRC-3 (B) mice. **A** eight-week-old male SRC-3$^{-/-}$ApoE$^{-/-}$ and SRC-3$^{+/+}$ApoE$^{-/-}$ mice were fed with WD for 12 weeks. **B** ApoE$^{-/-}$ mice were infected with endothelial-specific RGDKRVS-AAV9-shSRC-3 or shCtrl virus through tail vein injection before a WD feeding. *P* values were calculated by unpaired two-tailed Student's t-test. *, *P*<0.05; **, *P*<0.01.

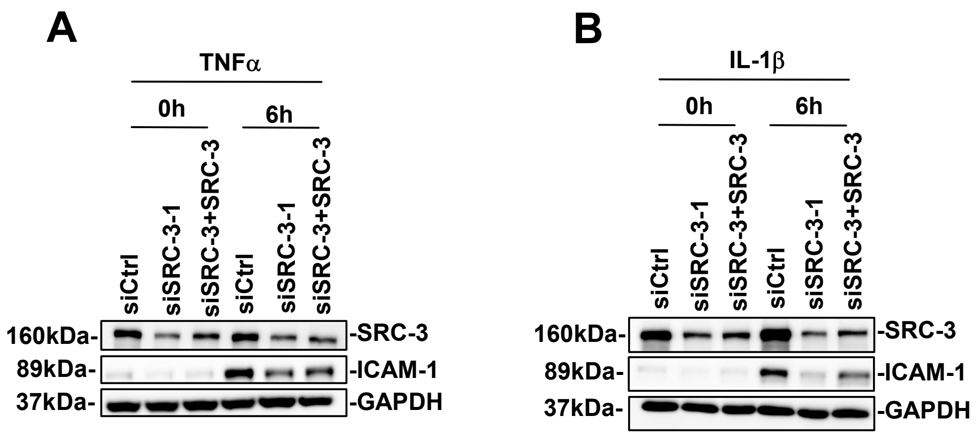

Supplementary Figure 2. Restoration of SRC-3 expression in SRC-3-knockdown HUVECs rescues ICAM-1 expression. A and B Restoration of SRC-3 expression in SRC-3-knockdown HUVECs could rescue ICAM-1 expression after TNFα (A) or IL-1β (B) treatment.

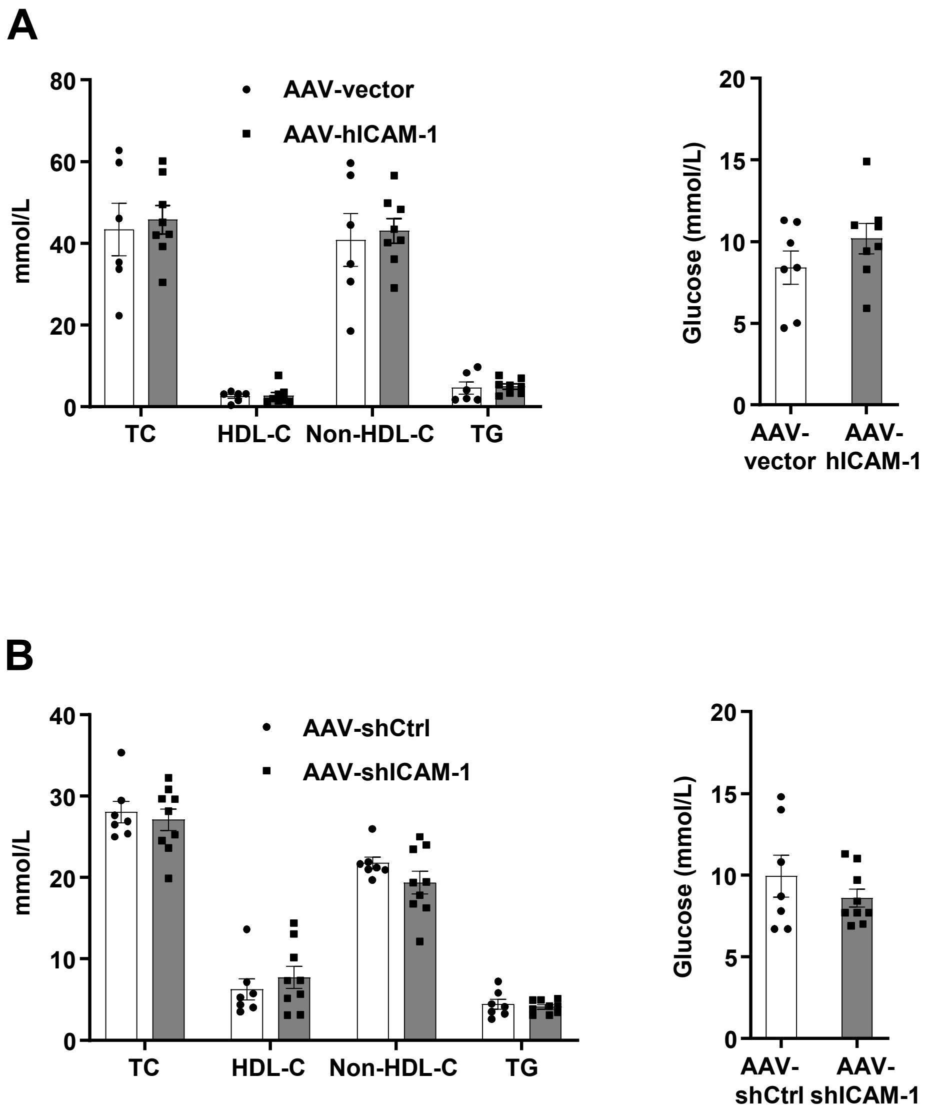

Supplementary Figure 3. Lipid levels and plasma glucose in AAV-hICAM-1 (A) or AAV-shICAM-1 (B) mice. A ApoE$^{-/-}$ mice were infected with endothelial-specific RGDKRVS-AAV9-ICAM-1 or -scramble virus through tail vein injection before a WD feeding. **B** ApoE$^{-/-}$ mice were infected with endothelial-specific RGDKRVS-AAV9-shmICAM-1 or -scramble virus through tail vein injection before a WD feeding.

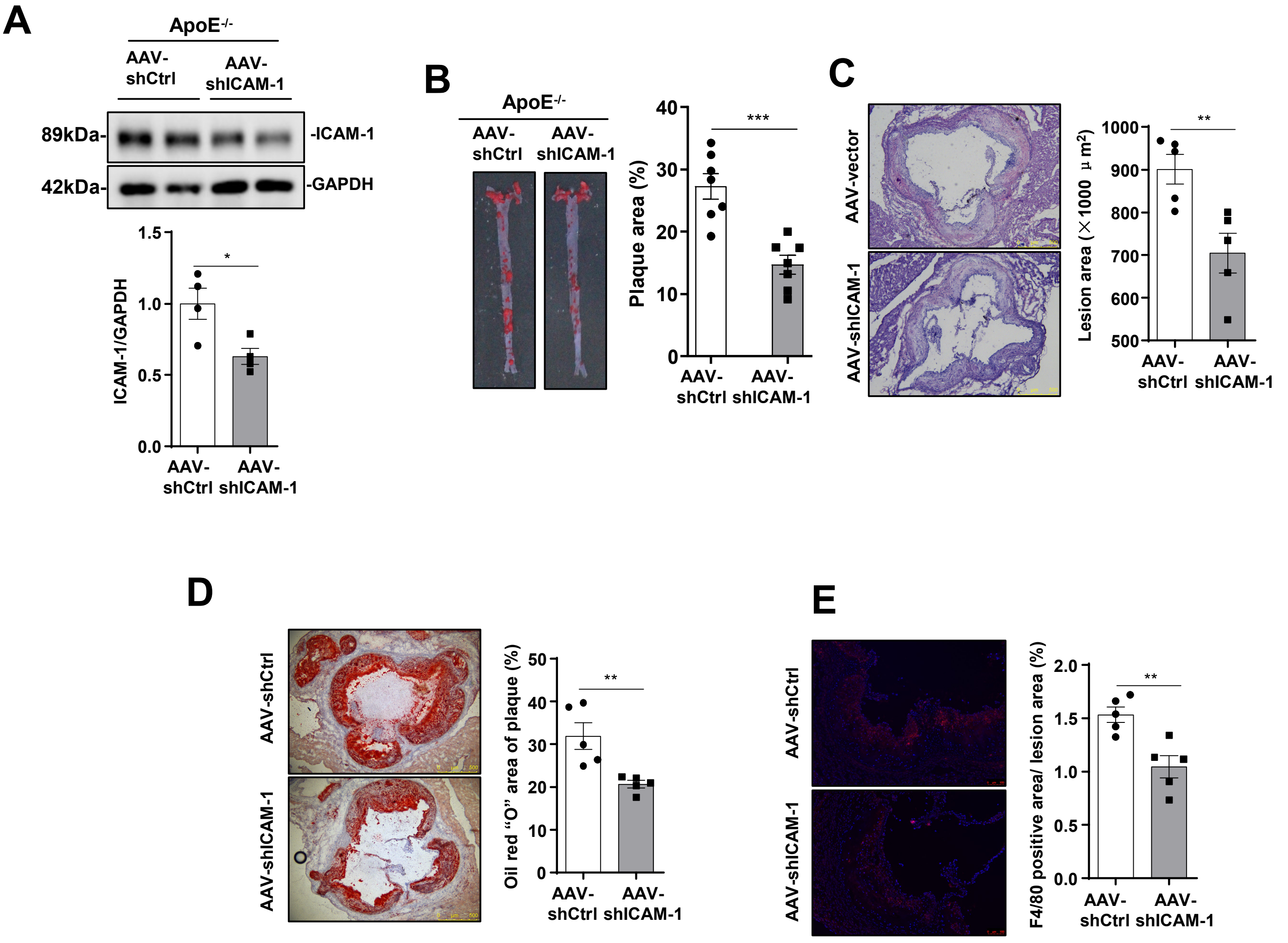

Supplementary Figure 4. Knockdown of ICAM-1 decreases atherosclerotic plaque formation and lesion area.
A Western blot showing that AAV-mediated ICAM-1 shRNA reduced ICAM-1 expression levels in the aortas of ApoE$^{-/-}$ mice. **B** ICAM-1 knockdown decreased atherosclerotic plaque formation in ApoE$^{-/-}$ mice. Representative images of *en face* Oil Red O-stained aortas from ApoE$^{-/-}$ mice injected with AAV-mediated ICAM-1 shRNA and scramble shRNA (left panel). Quantification of the plaque areas of aortas (right panel). **C-E** Cross-sections of the aortic roots from ApoE$^{-/-}$ mice injected with AAV-mediated SRC-3 shRNA and scramble shRNA were subjected to (C) H&E staining (scale bar, 100 μm), (D) Oil Red O staining (scale bar, 100 μm), (E) F4/80 staining (scale bar, 100 μm). Left panels, representative images. Right panels, quantification of stained area or a percentage of lesion area. The data represent the mean ± SEM. The results are representative of three independent experiments. P values were calculated by unpaired two-tailed Student's t-test. *, P<0.05; **, P<0.01;***, P<0.001.

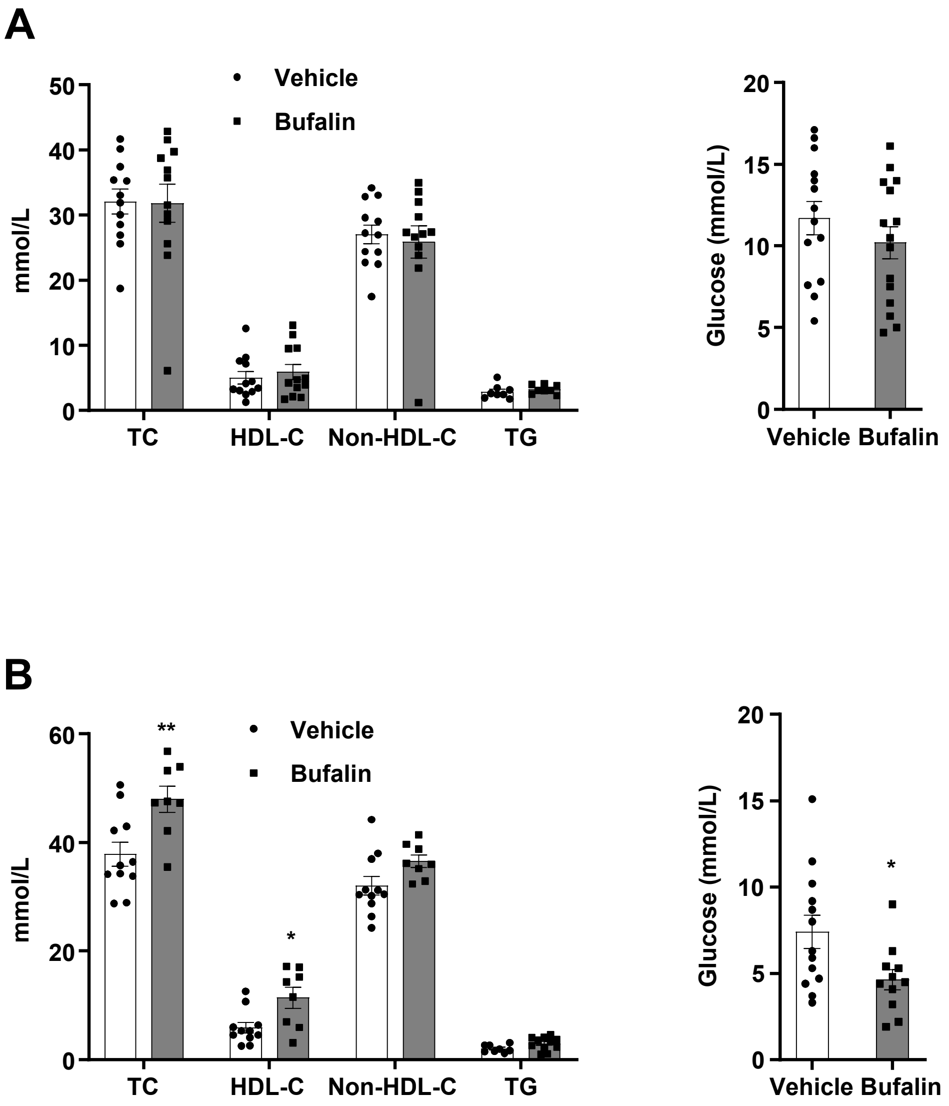

Supplementary Figure 5. Lipid levels and plasma glucose in bufalin-treated mice. A ApoE$^{-/-}$ mice were administered vehicle or bufalin (1.0 mg/kg, six times a week) by intraperitoneal injection for 13 weeks concomitant with a WD. **B** ApoE$^{-/-}$ mice were fed a WD for 10 weeks and then were treated with vehicle or bufalin (1.0 mg/kg, six times a week) by intraperitoneal injection for 13 weeks concomitant with a WD. *P* values were calculated by unpaired two-tailed Student's t-test. *, $P<0.05$; **, $P<0.01$.

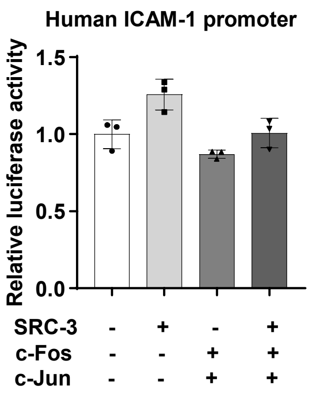

Supplementary Figure 6. SRC-3 can not cooperate with AP-1 to induce ICAM-1 promoter activity.

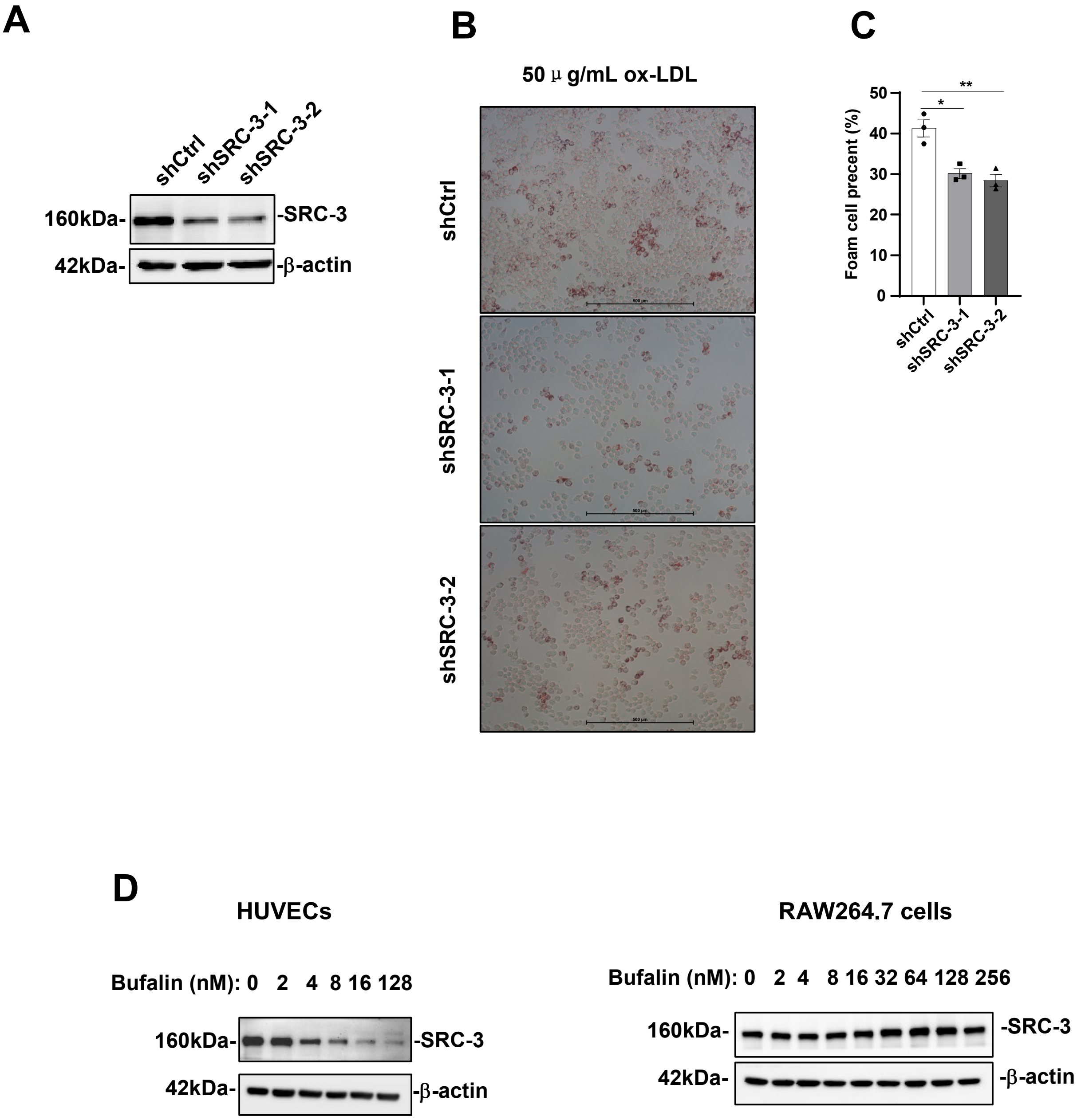

Supplementary Figure 7. SRC-3 knockdown reduces macrophage foam cell formation. A SRC-3-knockdown stable RAW264.7 cell lines. **B** and **C** SRC-3 knockdown reduced macrophage foam cell formation. **D** bufalin treatment didn't decreased SRC-3 expression in macrophages, while bufalin treatment could significantly reduce SRC-3 expression in HUVECs. The data represent the mean ± SEM. The results are representative of three independent experiments. *P* values were calculated by unpaired two-tailed Student's t-test. *, *P*<0.05; **, *P*<0.01.

Supplementary Table

Human clinical data

Arteriosclerosis obliterans Patient data			
**Number**	**Gender**	**Age**	**surgery**
1	Male	82	Right lower extremity amputation
2	Male	81	Right lower extremity amputation
3	Male	72	Right lower extremity amputation
4	Male	79	Right lower extremity amputation
5	Female	90	Left lower extremity amputation
6	Male	69	Left lower extremity amputation
7	Female	69	Left lower extremity amputation
8	Female	79	Right lower extremity amputation
9	Male	69	Left femoral artery endarterectomy
10	Female	80	Right lower extremity amputation
11	Male	67	Right femoral artery endarterectomy
12	Male	55	Right femoral artery endarterectomy
13	Female	81	Left femoral artery endarterectomy
14	Male	71	Right lower extremity thrombectomy
15	Female	77	Right lower extremity amputation
16	Female	79	Left lower extremity amputation
